# Observation of resonant exciton and correlated plasmon yielding *correlated* plexciton in amorphous silicon with various hydrogen content

**DOI:** 10.1038/s41598-022-24713-5

**Published:** 2022-12-13

**Authors:** Soni Prayogi, Retno Asih, Budhi Priyanto, Malik A. Baqiya, Muhammad A. Naradipa, Yoyok Cahyono, Darminto, Andrivo Rusydi

**Affiliations:** 1 Department of Physics, Institut Teknologi Sepuluh Nopember, Surabaya, 60111 Indonesia; 2 Department of Electrical Engineering, Pertamina University, Jakarta, 12220 Indonesia; 3 Department of Physics, National University of Singapore, Singapore, 117542 Singapore; 4 Singapore Synchrotron Light Source, 5 Research Link, Singapore, 117603 Singapore

**Keywords:** Materials science, Optics and photonics, Physics

## Abstract

Hydrogenated amorphous silicon (a-Si: H) has received great attention for rich fundamental physics and potentially inexpensive solar cells. Here, we observe new resonant excitons and correlated plasmons tunable via hydrogen content in a-Si: H films on *Indium Tin Oxide* (ITO) substrate. Spectroscopic ellipsometry supported with *High Resolution-Transmission Electron Microscopy* (HR-TEM) is used to probe optical properties and the density of electronic states in the various crystallinity from nano-size crystals to amorphous a-Si: H films. The observed optical and electronic structures are analyzed by the second derivative with analytic critical-point line shapes. The complex dielectric function shows good agreement with microscopic calculations for the energy shift and the broadening inter-band transitions based on the electron–hole interaction. Interestingly, we observe an unusual spectral weight transfer over a broad energy range revealing electronic correlations that cause a drastic change in the charge carrier density and determine the photovoltaic performance. Furthermore, the interplay of resonant excitons and correlated plasmons is discussed in term of a correlated plexciton. Our result shows the important role of hydrogen in determining the coupling of excitons and plasmons in a-Si: H film for photovoltaic devices.

## Introduction

Hydrogenated amorphous silicon (a-Si: H) has recently emerged as a favorite material for making large-area thin film-based optoelectronic devices such as thin film solar cells^1^, radiation detectors^2^, image sensors^3^, thin film transistors^4^, memory devices^5^, and microchannel plates on both rigid^6^ and flexible substrate^7^. Besides being inexpensive, environmentally friendly, and non-toxic, this type of material is important because it can be n-type and p-type doped^8–10^ and the p–i–n homo junction structure has been realized without the band gap discontinuity at the interface^11–13^. Numerous studies have shown the presence of weak Si–Si bonds, Si–H_2_ bonds, and cavities that change a-Si: H optoelectronic properties^14–17^.

Electron–hole interactions, known as an exciton, play an important role in semiconductor and photovoltaic devices^18–20^. A dilution of hydrogen from the process gas mixture of silane and hydrogen has been used to improve the stability of a-Si: H materials and devices^21^. Recent results suggest that a significant increase in the stability of a-Si: H cell occurs when the intrinsic layer is created from a gas mixture diluted with hydrogen^22–24^. While the hydrogen content of this material is similar to alloys made with low hydrogen dilution, the effusion of hydrogen from this film occurs at a much lower temperature^25,26^. Besides, in the presence of electron excitation or thermal stimulation, the small mass and size of hydrogen atoms enable them to easily migrate within the relatively rigid Si matrix, resulting in some metastable structural evolution known as *Staebler Wronski Effect* (SWE)^27^. However, the role of hydrogen on electronic and optical properties in a-Si: H remains unexplored.

Here, we present a novel approach to generating amorphous structures of silicon from SiH_4_ with hydrogen dilution by the RF-PECVD technique and reveal the detailed evolution of the electronic structure in creating exciton and plasmon coupling and their relationship to the performance of photovoltaic devices. We obtain an accurate physical model of the optical response and structures of a-Si: H by using spectroscopic ellipsometry.

## Methods

The a-Si: H thin layer is intrinsically deposited on corning 1737 glass and ITO substrates employing the RF-PECVD, (MVSystem Inc. USA) technique in key-based UHV space loads with a key electrode area of 19.62 cm^2^ and 4 cm electrode separation. The total deposition time for each film is kept constant for 30 min. The deposition parameters for the dilution of hydrogen SiH_4_/H_2_ are 0, 16, and 36 using RF power of 10 W, substrate temperature of 270 °C, and process pressure (PP) of 2000 mTorr, as presented in Table 1. The R-0 film (without hydrogen dilution) deposition is carried out during precipitation, while the R-16 and the R-36 films are deposited with hydrogen dilution in 30 min. The atomic force microscope (Agilent 5500) is used to study the surface morphology and roughness of the films. The dark and photoconductivity of the films are measured under vacuum (∼ 10^−5^ mbar) in coplanar geometry, at the temperature range of 300–475 K, to estimate the activation energy.

**Table 1:** Deposition parameters for the (R-0 to R-36) layers used in solar cell.

Sample	Substrates	Hydrogen dilution (H_2_/S_i_H_4_)	Silane concentration (%)	Chamber pressure (mTorr)	Power RF (watt)	Substrates temperature (^o^C)	Deposition time (min)
R-0	ITO	0	100	2000	10	270	30
R-16	ITO	16	5.9	2000	10	270	30
R-36	ITO	36	2.7	2000	10	270	30

Spectroscopic ellipsometry parameters ψ and Δ (viz., the ratio of the amplitude and phase difference between p- and s-polarized reflected light, respectively) are collected at 50°, 60°, and 70° angles of incidence, with the photon energy range between 0.6 and 6.5 eV using the measuring apparatus (V-VASE, J. A. Woollam Co.) with a rotating analyzer and a compensator at the Singapore Synchrotron Light Source (SSLS). The measurements are performed in ultrahigh vacuum chamber, which allows a clean surface to be measured^28^ and a universal fitting is performed within the same optical parameters. Details of the instrument and the measurement geometry are described elsewhere^29^. The real and imaginary parts of the dielectric constant <ε_1_> and <ε_2_> are extracted using a least-squares regression analysis^30^ and an unweighted root-mean-square error function by fitting the experimental spectra with Woollam Complete Ease software. The parameters corresponding to the surface roughness of the R-0, R-16, and R-36 samples and the parameter of the respective dielectric functions are evaluated by a combination of the Tauc-Lorentz (TL)/Tauc-Lorentz + G model to determine thickness, band gap and optical constants of the films.

The following equations are used to evaluate the dielectric properties for the current set by a-Si: H^31^.

1. Complex dielectric function $\varepsilon \left(\omega \right)= {\varepsilon }_{1}\left(\omega \right)+i{\varepsilon }_{2}\left(\omega \right)$ (*ω* = angular frequency of the incident photon).
2. Refractive index $$n \left(\omega \right)=\sqrt{\frac{1}{2}[\sqrt{{\varepsilon }_{1}^{2}\left(\omega \right)+{\varepsilon }_{2}^{2}\left(\omega \right)}+{\varepsilon }_{1}\left(\omega \right)]}$$.
3. Extinction coefficient $$\kappa \left(\omega \right)=\sqrt{\frac{1}{2}[\sqrt{{\varepsilon }_{1}^{2}\left(\omega \right)+{\varepsilon }_{2}^{2}\left(\omega \right)}-{\varepsilon }_{1}\left(\omega \right)]}$$.
4. Loss-function $$-\mathrm{Im }\left[{\varepsilon }^{-1}\left(\omega \right)\right]=\frac{{\varepsilon }_{2}\left(\omega \right)}{[{\varepsilon }_{1}^{2}\left(\omega \right)+{\varepsilon }_{2}^{2}\left(\omega \right)]}$$.
5. Normal incident reflectivity $R \left(\omega \right)= \frac{{[n\left(\omega \right)-1]}^{2}+{\kappa }^{2}(\omega )}{{[n\left(\omega \right)+1]}^{2}+{\kappa }^{2}(\omega )}$.
6. Optical conductivity ${\sigma }_{1}\left(\omega \right)= {{\varepsilon }_{0}\varepsilon }_{2}\left(\omega \right) \omega$ (${\varepsilon }_{0}$ is the free-space permittivity).

For microscopy study using transmission electron microscope (TEM), all films are scratched and sonicated in acetone for several minutes and dispersed on a copper net. The high resolution- transmission electron microscope (HR-TEM) images and selected area electron diffraction (SAED) patterns are recorded using JEOL-2100.

## Results and discussion

**Figure 1:**
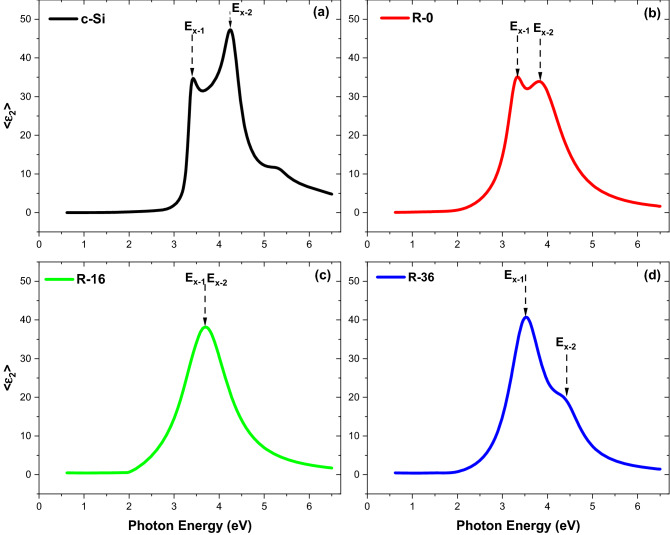
Imaginary part of complex dielectric function (E_x−1_: conventional exciton, E_x−2_: resonant excitons) <ε_2_> (**a**) c-Si, (**b**) R-0, (**c**) R-16, and (**d**) R-36.

Figure 1a shows that there are two exciton peaks, namely E_x−1_ = 3.4 eV and E_x−2_ = 4.24 eV with a wide energy shift between E_x−1_ and E_x−2_ of 0.84 eV originating from c-Si measurements using spectroscopic ellipsometry. For R-0 film in Fig. 1b, the exciton of both is pushed to the spectral weight at low energy and seen that there is a widening at <ε_2_> from E_x−1_ = 3.34 eV and E_x−2_ = 3.82 eV with a magnitude of energy shift of 0.48 eV, so that the quantum confinement effect occurs with the transmission changing from high energy, crossing zero to lower energy^32^. Furthermore, it is important that there is spectral weight transfer at <ε_2_> of the R-0 film, but is unseen in one of c-Si. It turns out that the peak in <ε_2_> of the c-Si film occurs at high energy, namely at E_1_ = 5.34 eV. There is a transfer of spectral to lower energy at E_0_ of 3.32 eV, which causes a spectral weight transfer of 3 eV, which is a signature of electronic correlation. Figure 1c depicts the <ε_2_> curve of the R-16 film, showing not only the quantum confinement effect, but also the dramatic effect of hydrogen. It is seen that both exciton peaks shift to each other and collap to be one exciton. When more hydrogen is added, as the R-36 film in Fig. 1d, there is another shift of 0.84 eV in the <ε_2_> curve between E_x−1_ = 3.52 eV and E_x−2_ = 4.36 eV. It leads to a rechanging exciton feature, which is somewhat the same as that of the c-Si film. This means that the hydrogen dilution has taken an important role in tunning the electronic structure of the deposited a-Si film.

**Figure 2:**
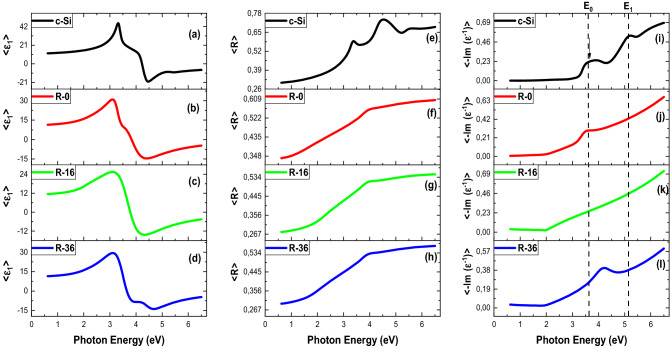
(**a–d**) Real part <ε_1_> of the dielectric function, (**e–h**) reflectivity, and (**i–l**) loss function (E_0_: conventional plasmon, E_1_: correlated plasmons) c-Si, R-0, R-16, and R-36.

Figure 2 displays the real part <ε_1_> of the complex dielectric function, loss function, and reflectivity of c-Si, R-0, R-16, and R-36 films. The value of <ε_1_> changes dramatically from positive to negative, showing different type of plasmons (Fig. 2a–d) as further discussed later. The <ε_1_> reaches a minimum with a positive value at about ~ 4.45 eV. For all films, <ε_2_> exhibits a peak at ~ 3.34 eV and the rise is a characteristic of silicon, featuring a resonant exciton. Resonant excitonic effects are typically occurred above the optical band gaps due to strong electron–hole and electron–electron interactions and have been observed in graphene^33–38^. The substrate also has non-reflective characteristics at ~ 1.00 eV but exhibits non-zero reflectivity at ~ 4.00 eV in Fig. 2e–h. When the a-Si: H film is deposited on the substrate, the reflectivity approaches zero and shows a depth at ~ 4.00 eV, shifting slightly with the addition of hydrogen. The minimum reflectivity at ~ 4.00 eV is a sign of plasmon.

The direct way to detect plasmons is via loss function^39^ as shown in Fig. 2i–l. The loss function shows two peaks, at ~ 3.50 eV and ~ 5.20 eV. By combining loss function and <ε_1_>, we identify two different types of plasmons. For the former where <ε_1_> is positive, it is unconventional, correlated plasmons^29^. For the later, where <ε_1_> is negative, it is conventional plasmons. The loss function curve does corroborate that the plasmon appears in the film^39^, characterized by a peak at ~ 3.50 eV in Fig. 2i–l. The peaks show the red and blue shifts of ~ 0.74 eV respectively for the R-0 and R-36 films, which are consistent with the deep reflectivity. Another peak is also observed at 5.20 eV, implying that the plasmon is coupling with the exciton at this photon energy.

The macroscopic linear optical response of Si is represented by the complex dielectric function $\varepsilon \left(\omega \right)$, which is closely related to the electronic band structure of the material. The structures observed in $\varepsilon \left(\omega \right)$ are attributed to interband transitions as well as excitons at critical points, which can be analyzed in terms of standard analytic line shapes: $\varepsilon \left(\omega \right)=C-A{e}^{i\phi }(\omega -E+i\Gamma {)}^{n}$, where a critical point (CP) is described by the amplitude *A*, threshold energy *E*, broadening Γ, and the excitons phase angle *Ф*. The exponent *n* has the value $-\frac{1}{2}$ for one-dimensional (1D), 0 logarithmic, i.e., $ln(\omega -E+i\Gamma$) for 2D, and $\frac{1}{2}$ for 3D CP's. Discrete excitons are represented by *n* =  − 1. The information obtained from the line-shape analysis can be compared with band structure calculations^40–42^.

**Figure 3:**
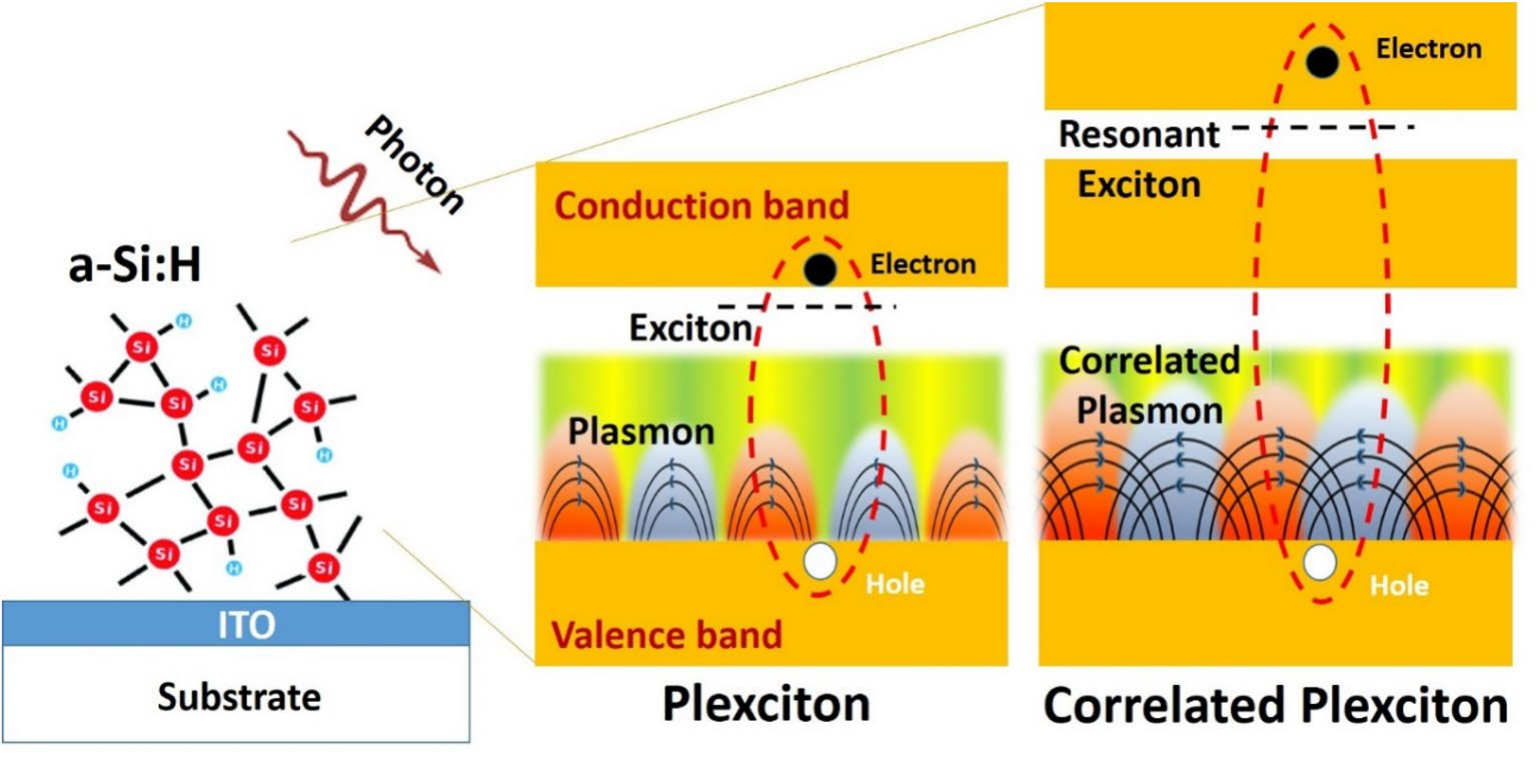
Schematic diagram of the coupling mechanism of correlated plasmon and resonant exciton.

It is interesting to further review the appearance of excitons and plasmons in the samples. In the c-Si film, two resonant excitons (Fig. 1) and two plasmons (Fig. 2) are observed, consisting of a correlated plasmon (lower energy) and a conventional plasmon (higher energy). The strong coupling between these two types of quasi-particles^43–47^, constitutes the so-called plexciton. Since the plexciton in this study is a coupling between resonant exciton and correlated and/or conventional plasmons, we propose that this is a *correlated plexciton*. The correlated plexciton structures are observed to be persisted despite the detailed structure gradually changes in a-Si and a-Si: H, suggesting the importance of electronic correlations. As can be seen in Fig. 1, the two resonant excitons (E_x1_ and E_x2_) undergo a shift in its energy closer to each other (R-0), merging into one exciton (R-16) and recover into two excitons again (R-36) in the presence of an increasing number of H atoms as illustrated in Fig. 3. On the other hand, the plasmons are still present in the whole a-Si samples, therefore, indicating that the presence of correlated plexciton could be confirmed.

**Figure 4:**
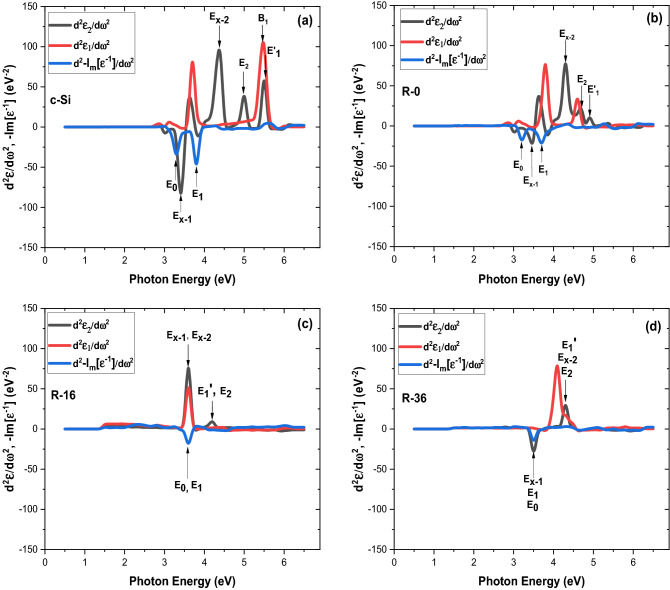
Fits to the second derivatives of the real (blue line) and imaginary (red line) parts complex dielectric function, include loss function of (**a**) c-Si, (**b**) R-0, (**c**) R-16, and (**d**) R-36.

To enhance the structure in the spectra and to perform a line-shape analysis of the CP, we calculate numerically the second derivative of the complex dielectric function with respect to photon energy d^2^ε/dω^2^, as presented in Table 2. Figure 4 shows the experimental second-derivative spectrum of <ε_1_>, <ε_2_>, and loss function in the spectral regions where structures are observed (points). The solid and dotted lines represent the best fits to standard critical-point line shapes, derived from: $$\frac{{d}^{2}\varepsilon }{{d\omega }^{2}}=\left\{\begin{array}{*{20}l}n\left(n-1\right)A{e}^{i\Phi }(\omega -E+i\Gamma {)}^{n-2}, n\ne 0\\ A{e}^{i\phi }(\omega -E+i\Gamma {)}^{-2}, n=0\end{array}\right.$$. The fit is performed simultaneously for the real and imaginary parts of d^2^ε/dω^2^ using a least-squares procedure^48,49^. If the angle Ф in the phase factor ${e}^{i\Phi }$ take values, which are integer multiples of π/2, the line shape corresponds to transitions between uncorrelated one-electron bands while noninteger multiples are usually believed to include excitonic effects by allowing a mixture of two CP integer multiple line shapes.

**Table 2:** *Critical-point* (CP) parameters used in the calculation of second derivatives.

CP parameter	c-Si	R-0	R-16	R-36
*E*_*x−*1_ (eV)	3.40	3.34	3.66	3.52
*E*_*x−*2_ (eV)	4.24	3.82	3.66	4.36
*E*_*o*_ (eV)	3.32	3.20	3.66	3.52
*E*_1_ (eV)	3.80	3.76	3.66	3.52
*E*_2_ (eV)	4.60	4.70	4.20	4.36
*E*_1_*ʹ* (eV)	5.34	4.95	4.20	4.36

**Figure 5:**
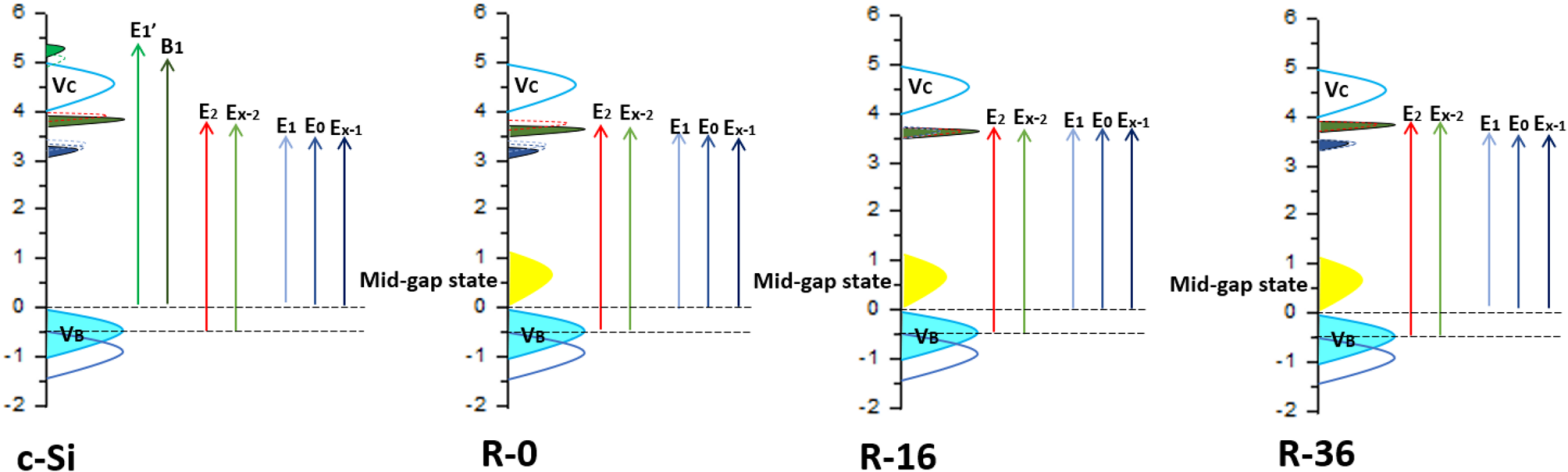
Schematic diagram of electronic band-structure in c-Si, R-0, R-16, and R-36.

Figure 5 shows a possible band scheme, where the valence band is positioned at 0 eV and the conduction band is positioned at ~ 4.00 eV. This placement is based on the assumption that the conduction band dissects the gap between the highest charged state and the lowest empty state (this gap is essentially the initial energy for the charge transfer process) in a 1:3 ratio, as seen from recent reports on c-Si. This estimation for the valence band position (i.e., 0 eV and below) is consistent with the < 1 eV gap between the valence band and conduction band in another recent calculation for c-Si^50^. Based on the results of SE and second derivative analysis on the samples of c-Si, R-0, R-16 and R-36 that, at room temperature, a new midgap state is formed on the addition of H as illustrated in Fig. 5. Taking into account the fact that the system is not an ordinary semiconductor, we propose two new midgap states: one in the filled state and the other in the empty state with a very small finite gap in between. From the detailed observation of the electronic band structure, we imply that the unfilled middle gap state is likely to be formed by higher energy unfilled states, which fill the lower part of the conduction band. The proposed band structure also shows that, with the addition of H, the charge transfer transition tends to dominate the electron–hole transition, which is quantitatively confirmed by the spectral weight transfer^51^.

**Figure 6:**
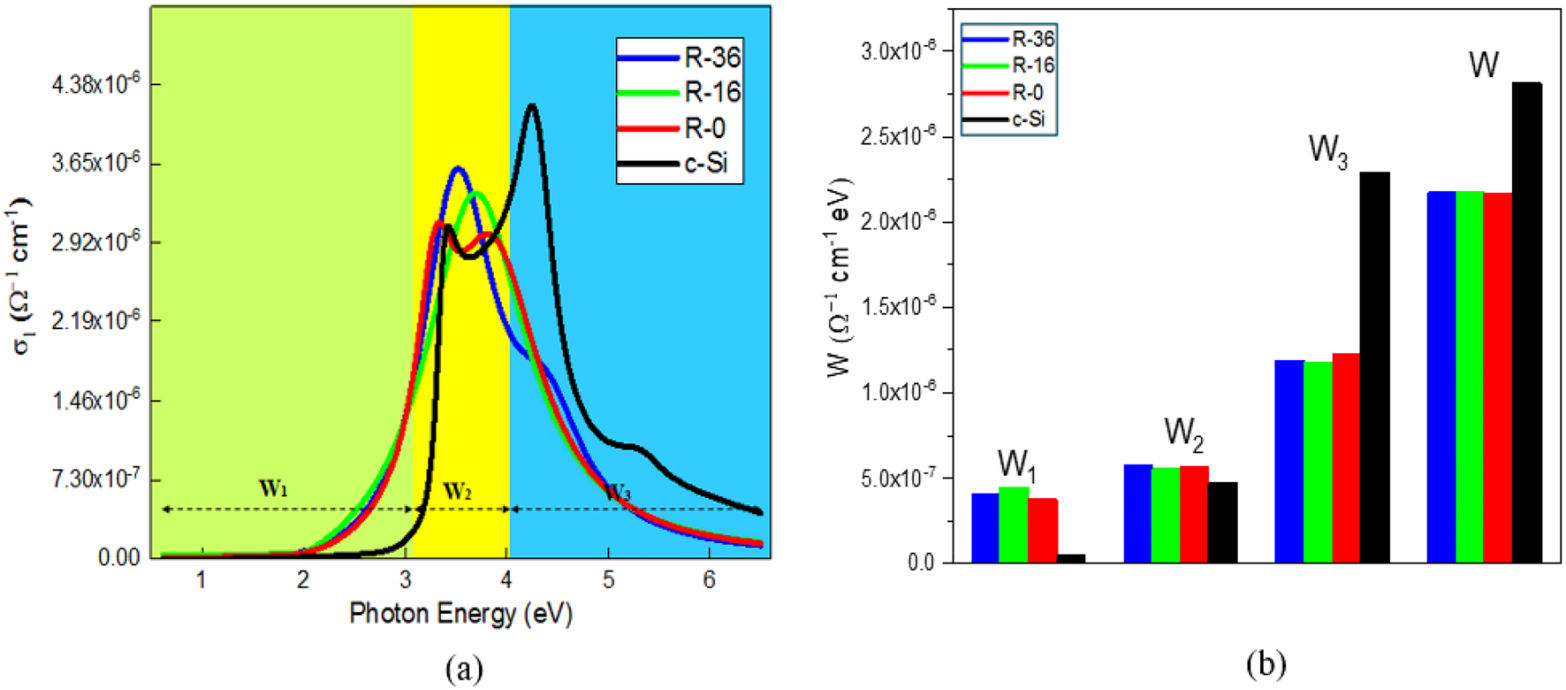
(**a**) Optical conductivity (*σ*_1_) spectra, and (**b**) spectral weight of R-0, R-16, R-36 and c-Si. Inset: The estimated spectral weight transfer over three energy ranges: 0.6–3.1 eV (*W*_1_), 3.1–4.0 eV (*W*_2_), and 4.0–6.5 eV (*W*_3_), while *W* is defined as *W*_1_ + *W*_2_ + *W*_3_.

Since we are able to identify the optical transitions in the three a-Si: H, we can quantify the change in the optical transitions associated individually*.* This change is established quantitatively using the optical conductivity, *σ*_1_(*ω*) of the a-Si: H because *σ*_1_(*ω*) satisfies the *f*–sum (charge conservation) rule and is related to the total electron density *n* by the relation ${\int }_{0}^{\infty }{\sigma }_{1}$(*ω*) *dω* = *πne*^2^*/*2*m*_*e*_, where *m*_*e*_ is the rest mass of the electron^52^. For a finite-energy range, the integral can be expressed as *W* = ${\int }_{E1}^{E2}{\sigma }_{1}$(*E*) *dE*. The *W* is termed as the spectral weight transfer and is proportional to the *effective* number of electrons participating in the optical transitions within the energy range [E_1_, E_2_]. Considering the experimental energy range from 0.6 to 6.5 eV of our observed spectra, we divide the SWT into three different ranges, viz., *W*_1_ for energy range 0*.*6–3.1 eV, *W*_2_ for 3.1–4.0 eV, and *W*_3_ for 4.0–6.5 eV. Estimated *W*_1_, *W*_2_, and *W*_3_ and their sum *W* for each of the a-Si: H are shown in Fig. 6.

The total spectral weight W, from a-Si: H across the measured spectral range from 0.6 to 6.5 eV is shown in Fig. 6b. There is an increase in W when the sample adds hydrogen from R-16 to R-36, followed by a decrease to roughly the same level as in R-0 when the sample is missing hydrogen. These results indicate that there are more electrons with energies between 0.6 and 6.5 eV on R-36 than in R-0 and R-16. This means that in the R-36 sample, the number of electrons with energies beyond the measured spectral range increases. This spectral weight shift cannot be explained by the activation of the addition of a hydrogen atom only, because the energy associated with R-0 and R-16 is too small (< 33 meV); therefore, any extra energy gained or lost must come from the electron–electron correlation potential energy. Both plasmon and exciton couplings are seen in the R-0, dissipating at the R-16, which also coincides with the decrease in electron conductivity and electron density. Figure 6b shows the change in W for each of the three spectral regions of Fig. 6a when the sample adds hydrogen from R-16 to R-36. While there is little change in the low and medium energy regions, the spectral weight loss in the high-energy regions shows a significant shift in electron density from this spectral range to higher energies (above 6.5 eV). The increase in electron energies on the order of several eVs comes from the correlation of the long-distance electrons, which is now prominent due to decreased electronic filtering^53^. This in turn gives rise to plasmonic activity, which is seen in conductive materials.

**Figure 7:**
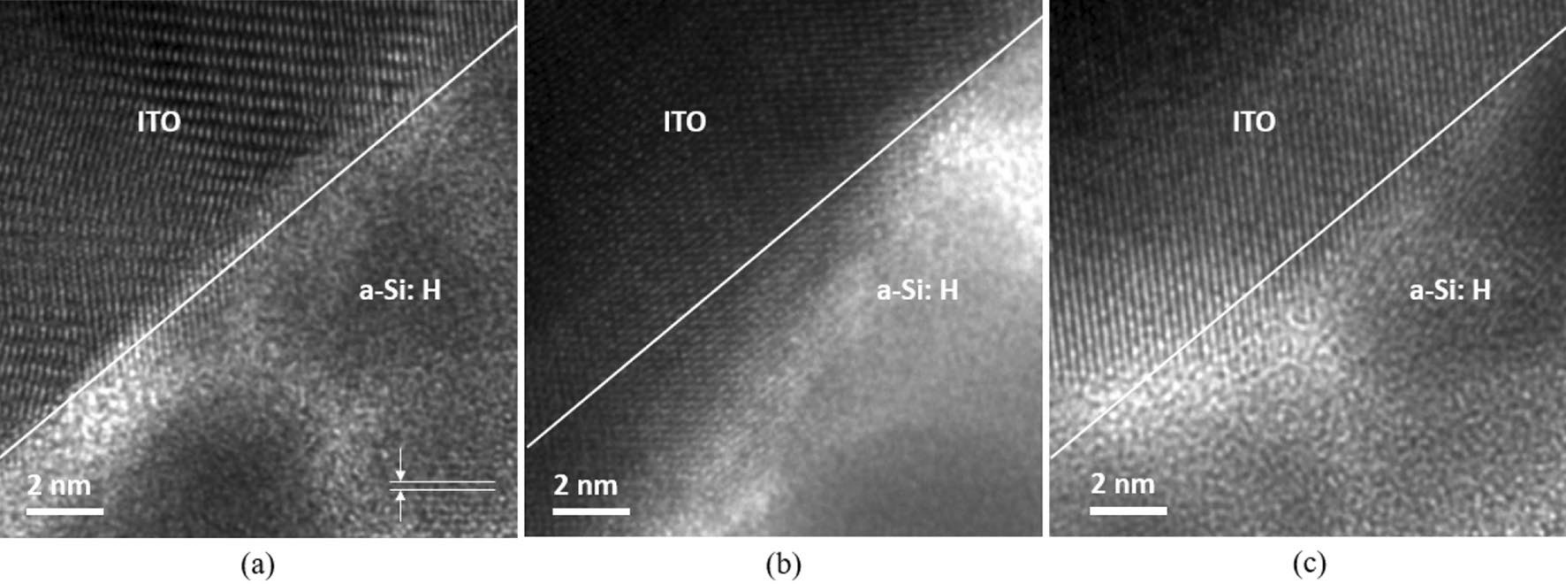
HR-TEM images of (**a**) R-0, (**b**) R-16, and (**c**) R-36 films, showing an evolution from nanocrystalline regime to amorphous phase due to hydrogen dilution.

Given in Fig. 7 is the HR-TEM images of the films with different hydrogen content. A crystalline island having size of several nm can be observed in the R-0 film (Fig. 7a), exhibiting a strong proof in a nanocrystalline regime (nc-Si: H). The porous structure of the R-0 film is caused by the H_2_ etching effect of the plasma. The inter-planar distance (d) calculated from the image is between 0.2 and 0.3 nm, corresponding to (111) and (220) planes in Si crystal. This can be attributed to the increased bonding and microstructure of these films subjected to hydrogen plasma. During the hydrogen treatment, a large flux of hydrogen atoms arriving at the surface of the film breaks the weak Si–Si bond and replaces it with a strong Si–Si bond^54^. The hydrogen atoms also diffuse into most films and improve the structure of the film^55^. During subsequent growth, Si atoms that arrive at the surface prefer to maintain this structural arrangement rather than forming a random network^56^. This process continues for a few layers of atoms and a random network of Si atoms is then formed, which results in the growth of a-Si: H. For the R-16 in Fig. 7b, the film is only subjected to hydrogen plasma treatment once and subsequent precipitation for a longer period, which is not resulting in changes in the film’s microstructure. For the R-36 film, with higher H_2_ dilution followed by shorter deposition time, the transformation from a-Si: H to nc-Si: H has resulted in a mixed phase of the amorphous and nano-crystalline structures, forming a denser amorphous film, as in Fig. 7c.

## Conclusions

In summary, by simultaneously measuring the complex dielectric function, loss function, and reflectivity of the hydrogen dilution effect in the a-Si films using spectroscopic ellipsometry, we determine electronic and optical structures and observe an unusual spectral weight transfer resulting high-energy resonant excitons and correlated plasmons and significant shifts in Fermi levels. From the spectral weight transfer analysis, we find that after addition of hydrogen, a decrease in electronic screening causes an increase in long-range electron correlation and increases the potential energy of the system resulting in the formation of resonant excitons and correlated plasmons couplings, namely a *correlated* plexciton. The scattering of electrons from the coupling of high-energy resonant excitons and correlated plasmons results in an increase in bulk carrier density and, subsequently, a reversible shift in Fermi energy. Overall, we believe that the findings of this work can help boost the power conversion efficiency of solar cells with the demonstration results of high-energy resonant excitons and correlated plasmons for the performance of photovoltaic devices in a-Si: H, and the methodology introduced here can be used to investigate excitons and plasmons in semiconductors and strongly correlated electron systems.

## Supplementary Information

Supplementary Information.

## Data Availability

All data generated or analyzed during this study are included in this published article [and its Supplementary Information files].
