# Supplementary material for: Observation of resonant exciton and correlated plasmon yielding correlated plexciton in amorphous silicon with various hydrogen content
Source: Sci Rep. 2022 Dec 13;12:21497. doi: 10.1038/s41598-022-24713-5 (PMC9748134; doi:10.1038/s41598-022-24713-5)
Supplement: Supplementary file 1 — Supplementary Information. [file 41598_2022_24713_MOESM1_ESM.docx]

**Supplementary information**


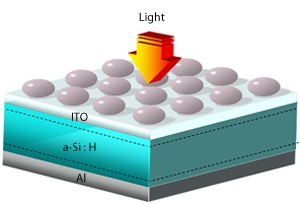

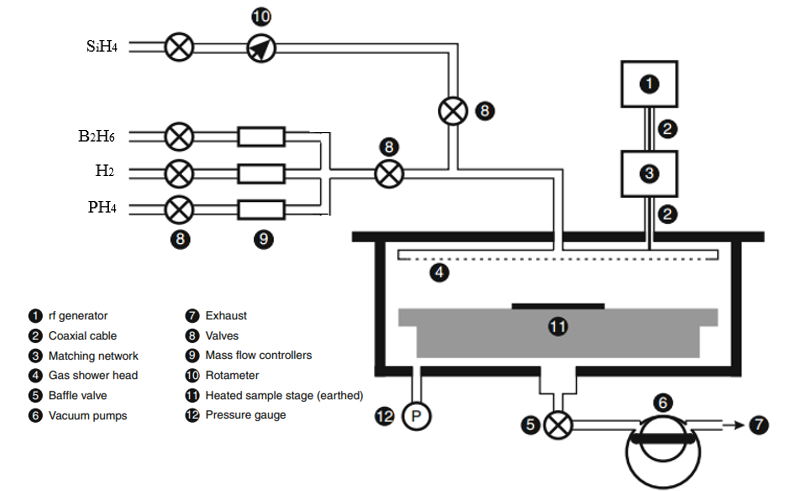


(a)

(b)

**Figura A-1**: Supplementary Figure. Sample preparation scheme a-Si: H solar cells. (a)Schematic RF-PECVD techniques and (b) Schematic diagram of the a-Si: H solar cells.

The a-Si: H thin layer is intrinsically deposited on corning 1737 glass and ITO substrates that do not use radio frequency plus plasma waves plus chemical vapor chemical deposition (RF-PECVD) techniques in key-based UHV space loads with a key electrode area of 19.62 cm^2^ and 4 cm electrode separation. The total deposition time for each film was kept constant at 30 minutes and 60 minutes. The deposition parameters for the dilution of hydrogen SiH_4_/H_2_ are 0, 16 and 36, RF power 10 W, substrate temperature 270°C, process pressure (PP) 2000 mTorr. For R-0 no hydrogen dilution was carried out during precipitation, while for R-16 and R-36 hydrogen were diluted for 30 minutes and 60 minutes as shown in Table 1. No plasma treatment is carried out on the top surface of the film. The scheme of precipitation and dilution of hydrogen for the sample is shown in Fig A-1.


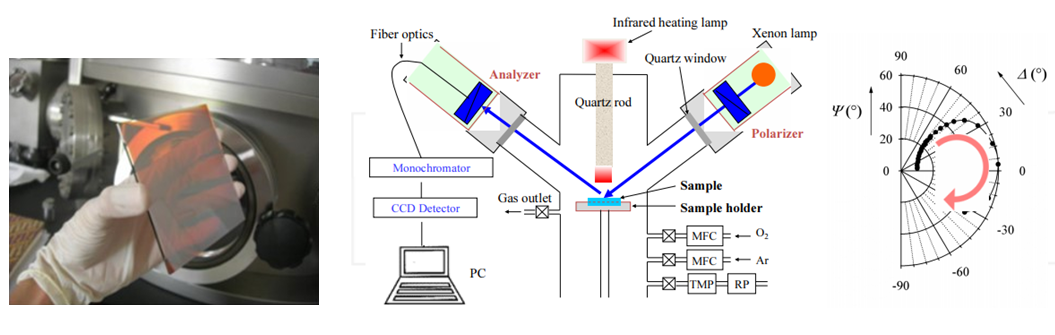


(a)

(b)

(c)

**Figura A-2**: Supplementary Figure. (a) Samples the amorphous panels (10x10) cm^2^ are integrated with conductive films. (b) Schematic diagram of the spectroscopic ellipsometric we designed, and (c) an example of the observed values of (Ψ and 𝚫).

During SiH_4_ plasma, a number of reactive species such as SiH_3_, SiH_2_, SiH, Si, H_2_ and H are produced which undergo secondary reactions, mostly with SiH_4_ parent molecules, forming a stable state. Each secondary reaction has a different rate constant. Highly reactive species like SiH_2_, SiH and Si have much less density in the plasma compared to SiH_3_ and thus SiH3 is the main film forming precursor on the substrate. The SiH_3_ radical reaching the substrate starts to diffuse on the surface. The growing films have a large density of defects like dangling bonds and weakly bonded Si-H_2_ bonds along with strong Si-H bonds. During H_2_ dilution, the films are exposed to large flux of atomic hydrogen, which can diffuse on the surface as well in to the bulk of the films and a number of reactions takes place. These include the passivation of dangling bonds, breaking of weak Si-Si and Si-H_2_ bonds at surface and in bulk replacing these by strong Si-Si and Si-H bonds and chemical annealing. This results in the top layers of growing films having less disorder and higher density. The H_2_ dilution on a-Si: H films can form the nc-Si: H films by chemical annealing process. This gives the crystallization of a-Si: H films through formation of systematic and ordered structure of Si network without removing Si atoms.

**Figura A-3**: Supplementary Figure. Experimental (solid curves) and fitted (dotted curves) data of Ψ and 𝚫 for R-0 (b, f), R-16 (c, g), and R-36 (d, h) obtained at 50^o^, 60^o^, and 70^o^

Experimental ψ and 𝚫 for R-0, R-16, R-36 and c-Si are shown in Fig A-3 together with the fittings from their analysis. To substantiate the formation of the excitation, we show, in Fig. 2, the loss function, reflectivity R, and the real part ε_1_ and imaginary part ε_2_ Fig. 1 of the complex dielectric functions derived using the spectroscopic ellipsometric data for the three samples, R-0, R-16, R-36 and c-Si collected at room temperature in the optical energy range of 0.6–6.6 eV.

**Table A-1**. Model fitted parameters (A, E_0_, C and Eg) of R-0 to R-36 films.

| **Sample** | **TL + G Model*/ TL model*** | | | |  | **TL + BEMA Model** | | | |
| --- | --- | --- | --- | --- | --- | --- | --- | --- | --- |
|  | **A**  **(eV)** | **E_o_**  **(eV)** | **C**  **(eV)** | **E_g_**  **(eV)** |  | **A**  **(eV)** | **E_o_**  **(eV)** | **C**  **(eV)** | **E_g_**  **(eV)** |
| **R-0** | 221.5 ± 1.32 | 3.45 ± 0.02 | 2.17 ± 0.,01 | 1.42 ± 0.02 |  | 225 ± 1.35 | 3.40 ± 0.01 | 3.5 ± 0.01 | 1.40 ± 0.01 |
| **R-16** | 225.7 ± 1.35 | 3.37 ± 0.01 | 2.14 ± 0.01 | 1.67 ± 0.01 |  | 225 ± 1.35 | 3.38 ± 0.01 | 3.5 ± 0.01 | 1.65 ± 0.01 |
| **R-36** | 230.4 ± 1.28 | 3.42 ± 0.01 | 2.26 ± 0.01 | 1.95 ± 0.01 |  | 230 ± 1.20 | 3.40 ± 0.01 | 3.2 ± 0.01 | 1.94 ± 0.01 |

The measured values ​​of the dielectric constant as a function of photon energy are mounted using TL + BEMA ie the parameter values ​​mounted A, E_0_, C and Eg are listed in Table A-1 and the corresponding thickness, band gap and bias values ​​(at 632 nm) are listed in Table A-2. Values ​​calculated from the amorphous, crystalline and void fractions for the bulk and rough surface layers are listed in Table A-2 along with the roughness values ​​obtained from AFM. As seen from this table, there is a good agreement between the values ​​of A, E0, C and Eg between the two models, (TL / TL + G and TL + BEMA). The value of the amplitude parameter (A) was found to increase and the expansion parameter (C) decreased with increasing plasma H_2_ dilution (Table A-1). This observation shows an increase in structural orders with dilution of H_2_. It was also observed that the volume fraction of the amorphous and void phases decreased and the crystalline phase increased with increasing plasma hydrogen phase (Fig. 1). The thickness, band gap and refractive index (at 632 nm) calculated using the two models also agree with the values ​​obtained from the UV-VIS transmission data. The roughness of the film from the AFM study is also very similar to the thickness of the rough surface layer (Fig. A-4).

**Table A-2**. Thickness, void, amorphous and crystalline fraction of bulk and surface roughness layer of R-0 to R-36 films calculated from SE and RMS roughness from AFM measurements.

| **Sample** | **Bulk layer** | | | |  | **Surface roughness layer** | | | |  | **AFM** |
| --- | --- | --- | --- | --- | --- | --- | --- | --- | --- | --- | --- |
|  | **d_bulk_**  **(nm)** | **f_v_**  **(%)** | **f_a_**  **(%)** | **f_c_**  **(%)** |  | **d_surface_**  **(nm)** | **f_v_**  **(%)** | **f_a_**  **(%)** | **f_c_**  **(%)** |  | **Roughness (nm)** |
| **R-0** | 690 | 6.43 ± 0.12 | 42.57 ± 1.54 | 39.5 ± 2,72 |  | 9.55 | 13.4 ± 0.58 | 50.2 ± 2.01 | 39.4 ± 1.25 |  | 8.95 ± 0.2 |
| **R-16** | 612 | 8.32 ± 0.18 | 62.34 ± 3.01 | 16.7 ± 1,01 |  | 6.42 | 25.3 ± 0.65 | 63.8 ± 2.67 | 14.5 ± 1.08 |  | 6.20 ± 0.3 |
| **R-36** | 535 | 9.44 ± 0.22 | 83.06 ± 3.86 | 4.22 ± 0,28 |  | 3.68 | 31.4 ± 0.86 | 65.5 ± 2.88 | 4.10 ± 0.59 |  | 3.50 ± 0.2 |

Atomic force microscopy (AFM) measurements were performed using an instrument manufactured by AIST-NT (Smart SPM 1000). The instrument was used in tapping mode on a scanned area of 1 μm by 1 μm. The AFM images were evaluated applying several features of the Gwyddion software79 including data leveling, background subtraction and false color mapping. Figure 14 shows surface topographies of the samples with roughness values of 8.95, 6.20 and 3.50 nm. The root mean square roughness values for all the three cases are around 0.1 μm. Based on these result, the surface roughness was not modelled for the spectroscopic ellipsometric evaluations as a separate layer.


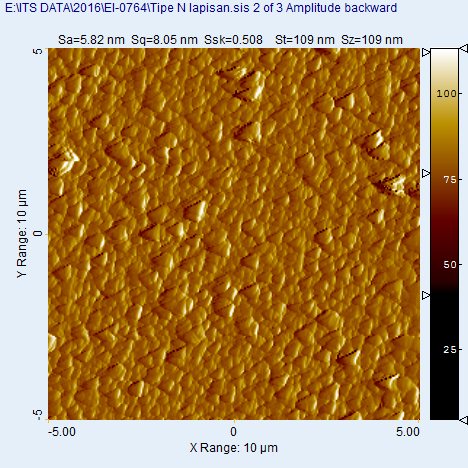

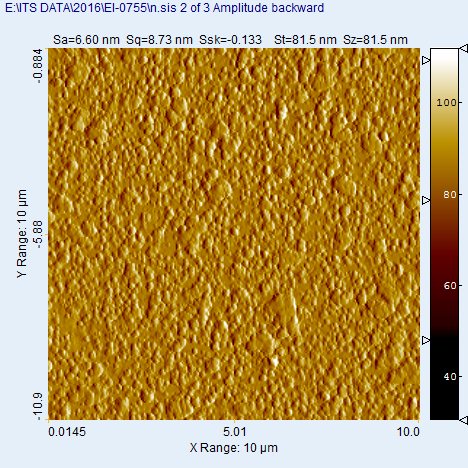

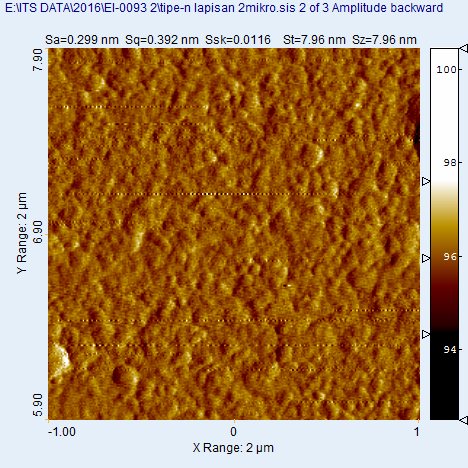


(a)

(b)

(c)

**Figura A-4**: Supplementary Figure. Line profiles and AFM topography images on the samples with (a) R-0, (b) R-16, and (c) R-36.
